# Environmental Impact of β-Cyclodextrin Complexes with Herbicides: A Study on Solubility and Toxicity

**DOI:** 10.3390/molecules31132361

**Published:** 2026-07-04

**Authors:** Gaetano Caputo, Elena Orlo, Roberta Nugnes, Chiara Russo, Martina Dragone, Gianluca D’Abrosca, Gaetano Malgieri, Carla Isernia, Margherita Lavorgna, Rosa Iacovino, Marina Isidori

**Affiliations:** 1 Department of Environmental, Biological and Pharmaceutical Sciences and Technologies, University of Campania “Luigi Vanvitelli”, Via Vivaldi 43, 81100 Caserta, Italy; gaetano.caputo@unicampania.it (G.C.); elena.orlo@unicampania.it (E.O.); martina.dragone@unicampania.it (M.D.); gaetano.malgieri@unicampania.it (G.M.); carla.isernia@unicampania.it (C.I.); margherita.lavorgna@unicampania.it (M.L.); marina.isidori@unicampania.it (M.I.); 2 Institute for the Study of Anthropogenic Impacts and Sustainability in the Marine Environment (IAS–CNR), 16149 Genova, Italy; roberta.nugnes@ias.cnr.it; 3 Department of Human Sciences, Link Campus University, Via del Casale di S. Pio V 44, 00165 Rome, Italy; g.dabrosca@unilink.it

**Keywords:** phase solubility diagrams, β-cyclodextrin inclusion complexes, rotifers, microalgae, chlorpropham, monuron, propanil, ecotoxicity

## Abstract

Formulation strategies that modify the physicochemical behavior of herbicides may influence their environmental fate and toxicity. In this context, this study investigates the effect of β-cyclodextrin (β-CD) complexation on the solubility and aquatic toxicity of chlorpropham (CLP), monuron (MON), and propanil (PRO), herbicides still in use in different parts of the world and frequently detected in aquatic environments at concentrations ranging from ng/L to µg/L. The solubility enhancement mediated by β-cyclodextrin was explored using UV-Vis and NMR spectroscopy, evaluating the impact of complexation on herbicides’ water solubility. Ecotoxicological evaluations were performed in *Raphidocelis subcapitata* (*R. subcapitata*) and *Brachionus calyciflorus* (*B. calyciflorus*), representing primary producers and consumers. Acute toxicity in *B. calyciflorus* significantly increased following complexation, with LC50 values decreasing from 178.09 µM (CLP), 32.32 µM (MON), and 20.77 µM (PRO) to 4.89, 2.55, and 2.29 µM, respectively. Chronic exposure further confirmed heightened sensitivity in rotifers (EC50: 0.04 µM for β-CD: MON; 0.02 µM for β-CD:PRO). *R. subcapitata* exhibited higher sensitivity to CLP (EC50: 2.57 µM), consistent with its mitosis inhibition mechanism. Risk Quotient (RQ) analysis, based on current environmental concentrations, revealed an ecotoxicological concern for MON and PRO. Overall, our study indicates that although β-cyclodextrin enhances herbicides solubility, it may also increase their bioavailability and toxicity underlining the necessity to evaluate a novel formulation not only from the point of view of the efficacy enhancement.

## 1. Introduction

Herbicides are primarily used for controlling weeds that compete with cultivated crops for essential resources such as light, water, nutrients, and physical space. Their mode of action involves disrupting critical physiological and biochemical processes in plants, such as photosynthesis, fatty acid synthesis, amino acid metabolism, or cell division, ultimately inhibiting growth or causing plant death [1,2]. All pesticides, including herbicides, play a crucial role in enhancing agricultural efficiency and boosting productivity. However, their extensive use has raised growing concerns regarding their potential impacts on surrounding ecosystems and biodiversity [3]. Although most herbicides are designed to act selectively on weeds, their limited specificity can lead to the unintended exposure of non-target organisms, since once applied, these compounds may migrate across various environmental compartments, ultimately resulting in the contamination of natural resources, including freshwaters [4]. This unintended exposure of these organisms can result in a range of harmful effects including behavioral alterations, reproductive impairments, immunosuppression, and neurological damage [5]. Furthermore, environmental processes such as drift and leaching facilitate the spread of these compounds beyond their intended targets, increasing overall exposure and amplifying ecological risk [6,7,8]. Consequently, herbicide contamination of surface and groundwaters ranging from nanograms to milligrams per liter has become an issue of global concern [9,10,11]. In response to the adverse effects associated with pesticide exposure, as well as their bioaccumulation and biomagnification along the food chain, both the scientific community and regulatory bodies have undertaken a critical reassessment of their use in order to limit potential risks not only to wildlife but also to human health. Over recent decades, this has led to increasingly stringent regulations and, in many cases, outright bans on several active substances in multiple countries. Nonetheless, it is important to recognize that pesticides have historically represented, and continue to represent, a fundamental tool for maintaining high crop yields and protecting plants from pests and diseases. Acknowledging their undeniable benefits, current research efforts are focused on the development of innovative and more sustainable strategies for pesticide use. The overarching goal is to maintain their effectiveness in pest control while minimizing the quantity of active ingredients required, thereby reducing environmental impact [12]. Among the promising approaches, the incorporation of pesticides into carriers at the molecular or nanoscale level represents an innovative strategy that improves their delivery to target organisms while reducing the required application concentrations [13].

**Figure 1:**
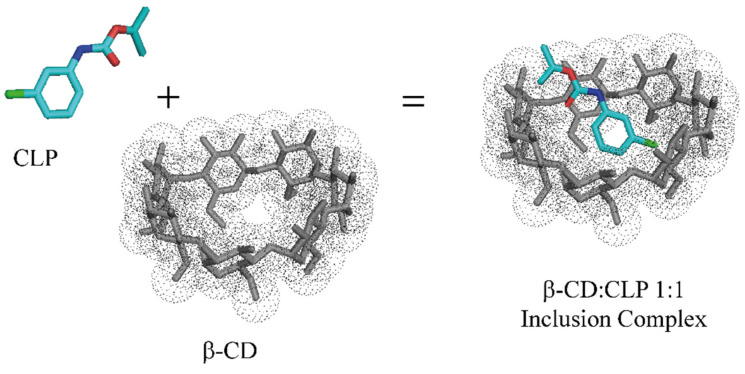
Scheme of the formation of β-CD: pesticide inclusion complex. The example reported is the formation of β-CD:CLP inclusion complex. The docked form is shown in a previous work by Dragone et al. 2023 [26].

One carrier system that has attracted significant attention is based on cyclodextrins (CDs), cyclic oligosaccharides composed of D-glucopyranose units [14,15]. Natural CDs are classified based on the number of glucose units they comprise: α-CD (six units), β-CD (seven units) and γ-CD (eight units). Owing to their cone-shaped structure, with an apolar interior and a hydrophilic exterior, β-CD can encapsulate hydrophobic pesticides, modifying their chemical-physical properties. In particular, β-cyclodextrins are low-cost and widely available compounds that can be exploited for the formulation for large agricultural use or other environmental applications. They can form inclusion complexes, as shown in Figure 1, with hydrophobic molecules [16,17,18]. The formation of these host-guest complexes not only enhances the water solubility of otherwise poorly soluble compounds, but can also influence their mobility, bioavailability, and environmental degradability [19,20], reduce the amount of pesticides needed, avoid their degradation in other more toxic compounds [21,22,23] and potentially decrease environmental impact [15,24]. However, lowering the amount of the pesticide used by means of novel and more effective formulations could result in an increase in terms of environmental toxicity that the overall beneficial ecological effect expected is lost. Thus, to ensure the safe and sustainable application of such advanced formulations, it is crucial to assess their potential environmental impact, particularly on non-target organisms [25]. Aquatic ecosystems are susceptible to contamination due to their direct exposure especially to agricultural runoff. In this context, ecotoxicological studies play a key role in both the development of pesticide carrier systems and the related risk assessment of them.

We have previously reported [26] the experimental and in silico physicochemical characterization, both in the solid state and in solution, of the interaction between β-cyclodextrins and three herbicides, chlorpropham (CLP), monuron (MON), and propanil (PRO), by means of X-ray powder diffractometry, UV-Vis and FT-IR. We showed that β-cyclodextrin is capable in all three cases of forming stable inclusion complexes with a 1:1 stoichiometry, with CLP, MON and PRO deeply embedded in the cyclodextrin cavity that shields their aromatic portions.

The present study complements the stoichiometry and structural characterization of these complexes with an accurate study of the solubility enhancement guaranteed by the complex formation [27]. We also consider that often the simple enhancement of solubility might involuntarily result in more ecotoxic formulations. In light of these considerations, we also conduct a comparative assessment of the aquatic toxicity of tested herbicides, both in their free form and as inclusion complexes with β-cyclodextrin (β-CD) (at a 1:1 molar ratio), and the evaluation of the environmental risk associated with the new formulations obtained.

These herbicides were selected based on their occurrence in surfaces and groundwater worldwide, with reported concentrations ranging from ng/L to µg/L [10,28,29], despite existing restrictions and bans implemented in various jurisdictions, particularly in Europe (Commission Implementing Regulation (EU) 2019/989; Regulation (EC) No 1107/2009; Directive 98/83/EC). These compounds target undesirable plant species through different mechanisms of action. MON and PRO disrupt photosynthesis by inhibiting electron transport within Photosystem II, effectively halting the energy production required for plant growth. CLP, on the other hand, impedes cell division by interfering with mitotic spindle formation, thereby inhibiting cellular growth and development.

Ecotoxicity tests were performed on two non-target freshwater organisms typical of two trophic levels: *Raphidocelis subcapitata*, a unicellular green alga (primary producer), and *Brachionus calyciflorus*, a planktonic rotifer (primary consumer). Both species are widely recognized as reliable sentinel organisms in ecotoxicological assessments due to their sensitivity to pollutants, ecological relevance, ease of cultivation, genetic uniformity, and rapid life cycles [30]. Their use allows the detection of impacts at both primary and secondary levels in the aquatic food web, especially in case of chronic and sub-lethal exposures. Therefore, acute toxicity (mortality) was evaluated in rotifers, while chronic toxicity (growth/reproductive inhibition) was assessed in algae and in rotifers after exposure to the tested substances.

The estimation of environmental risk was carried out in accordance with the European Environmental Risk Assessment (ERA) guidelines and the Technical Guidance Document (TGD, 2003).

## 2. Results and Discussion

### 2.1. Chemical Characterization

#### 2.1.1. Molar Absorptivity Determination

Our experimental results have examined and further elucidated the modification of guest physical-chemical properties: the formation of the cyclodextrin complex in solution has been utilized to enhance the solubility of herbicides in Milli-Q water.

**Figure 2:**
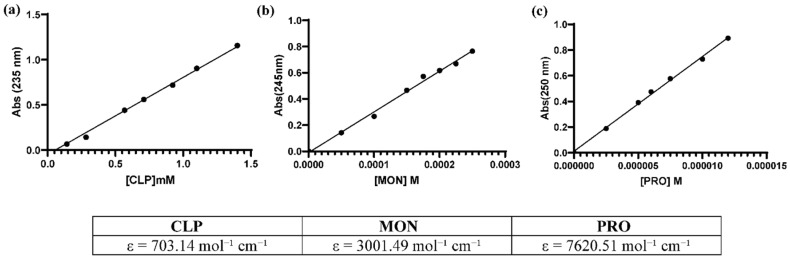
Calibration curve of CLP (**a**), MON (**b**) and PRO (**c**). In the table the value of absorption molar coefficient for each compound are reported.

The molar extinction coefficients reported in Figure 2 of CLP, MON, and PRO were determined by UV-Vis spectroscopy in accordance with the Lambert-Beer law. The peak intensity at 235 nm for CLP, at 245 nm for MON, and at 250 nm for PRO increases linearly corresponding to the increase in its concentration, allowing the calculation of molar absorptivity (ε). The slope of the absorbance-concentration curve corresponds to the molar extinction coefficient.

For each compound, seven measurements were performed at increasing concentrations. As the concentration of each herbicide increased, a corresponding rise in absorbance was observed at its characteristic wavelength. The average molar absorptivity (ε) was found to be 3001.49 mol^−1^ cm^−1^ for MON, 703.14 mol^−1^ cm^−1^ for CLP and 7620.51 mol^−1^ cm^−1^ for PRO.

#### 2.1.2. Phase Solubility Diagrams by UV-Vis

The chemical-physical characterization reported in Dragone et al. 2023 [26] of all inclusion complexes shows good stability with measured binding constants of 369.9 M^−1^ for β-CD:CLP, 292.3 M^−1^ β-CD:MON and 298.3 M^−1^ for β-CD:PRO. These findings, together with molecular docking simulations and job plot analysis, provide an accurate description of the inclusion properties of the cited complexes; however, they do not quantify the resulting solubility enhancement mediated by β-CD. Via UV-Vis spectroscopy, we carried out phase solubility studies measuring the change in the solubility of the guest compound as a function of the host concentration. The addition of β-CD led to a significant increase in the solubility of each compound. The phase solubility diagrams showed a positive correlation between cyclodextrin concentration and the amount of dissolved pesticide, further confirming the formation of the inclusion complexes.

**Figure 3:**
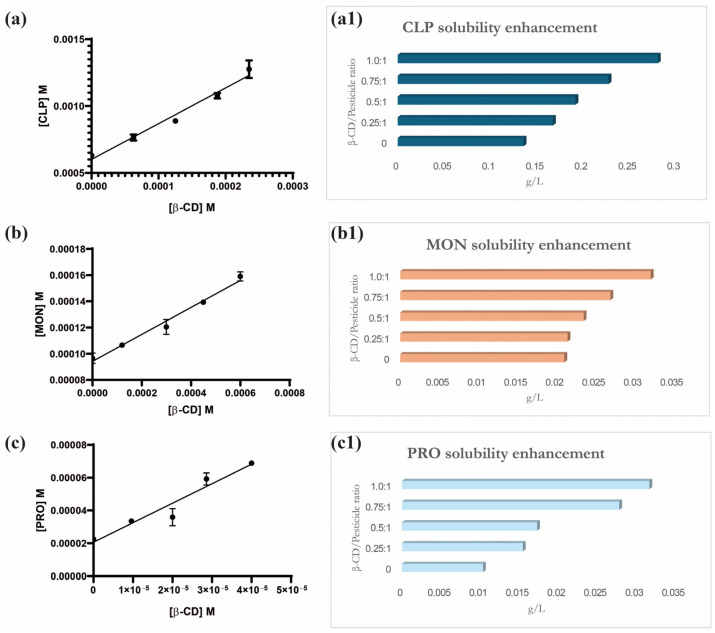
Phase solubility diagrams of CLP (**a**), MON (**b**) and PRO (**c**) in the presence of β-cyclodextrin. A linear increase in solubility is reported for each diagram. Bar graphs represent CLP (**a1**), MON (**b1**) and PRO (**c1**) solubility enhancement in buffered solution at pH 7.1 due to rising concentration of β-cyclodextrin.

The solubility diagrams (Figure 3a–c) of the phase of all the complexes are AL-type in agreement with the 1:1 molar ratio described in Dragone et al. [26]. The comparable magnitude of each K_b_ is in accord with the similar linear behavior in terms of solubility enhancement of the three inclusion complexes.

In Figure 3, panels a1, b1 and c1 show the bar graphs illustrating the solubility enhancement of each compound upon β-CD addition, in relation to the host-guest ratios reported on y-axis. As measured, PRO solubility increases approximately four-fold compared to its original limit; MON exhibits a doubled increase and CLP demonstrates a significant enhancement, with solubility improving by two times over its baseline value. These data confirm that such K_b_ values, which fall well within the range in which the compound is not too strongly (impeding the appropriate release of the guest) or too loosely bound, are enough to guarantee at least a two-fold increase in guest solubility, confirming the β-CD as an exploitable tool for novel herbicides formulations.

#### 2.1.3. Solubility Analysis by NMR

The increase in the solubility of the herbicides investigated in this study is also confirmed by NMR analysis. The samples prepared for the phase solubility diagrams analyzed via UV-Vis spectroscopy, were also studied by NMR in mono-dimensional ^1^H measurements.

The proton signals corresponding to each atom of the herbicides were assigned. They can be identified in the spectra recorded in the absence of cyclodextrin. Upon addition of cyclodextrin, the intensity of herbicides’ peaks, corresponding to protons directly involved in the inclusion process in the cyclodextrin cavity [26] increases. These data support the findings obtained via UV-Vis spectroscopy confirming a solubility enhancement for each β-CD:herbicide inclusion complex by a qualitative analysis.

**Figure 4:**
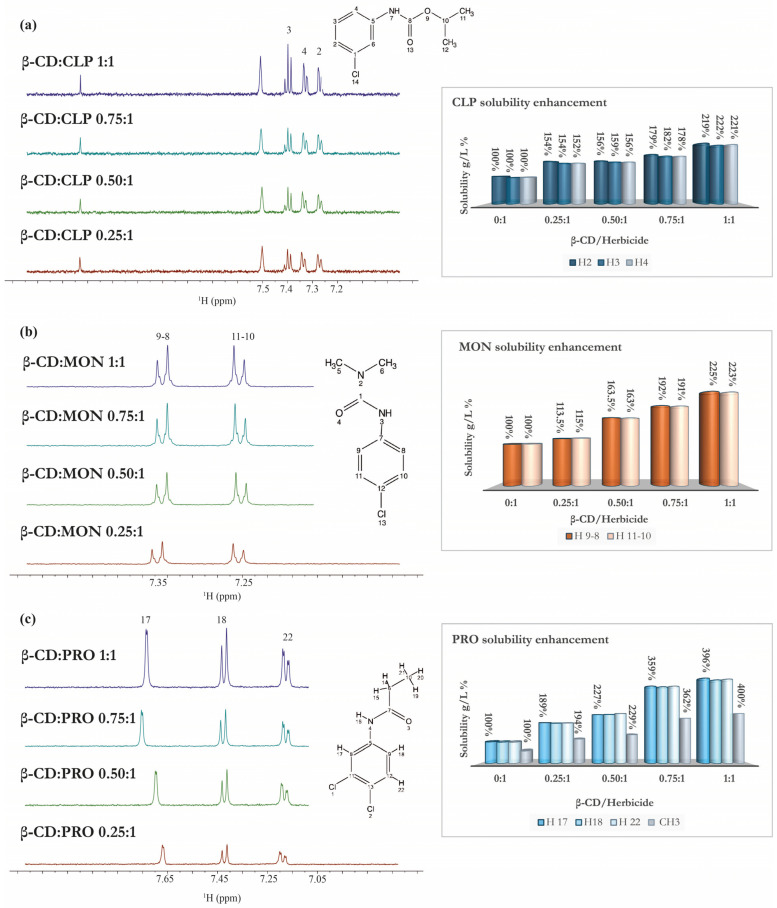
^1^H-NMR spectra relative at CLP (**a**), MON (**b**) and PRO (**c**) in different ratios with β-CD. The signals shown are those arising from the protons belonging to the aromatic rings of the three herbicides.

The spectra reported in Figure 4 show the aromatic protons of CLP (Figure 4a), MON (Figure 4b), and PRO (Figure 4c). The molar ratio between β-CD:herbicides starts from 0.25:1 in the spectrum on the bottom of the figure up to 1:1 in the upper spectrum.

All herbicide proton resonances were integrated and normalized against the relative signal intensity of the free compound (0:1 ratio, set as 100%). The increase in signal intensity reported as percentage ratio was used as a measure of the enhancement in apparent solubility induced by β-CD complexation. The proton signals mostly affected in terms of signal intensity (Figure 4) are the aromatic protons, which we have previously shown to be embedded into the cyclodextrin cavity.

### 2.2. Ecotoxicity

#### 2.2.1. Acute Toxicity Testing

Acute toxicity testing was conducted on *B. calyciflorus*, and lethality was recorded after 24 h of exposure to CLP, MON, and PRO, either in their free form or complexed with β-cyclodextrin. We have chosen to test the 1:1 molar ration on the basis of our phase solubility study. We acknowledge that the real-world formulations and environmental conditions may involve variable ratios; however, our choice yields the best possible situation to test the specific toxicity of the complex. Other ratios, also in light of the known chronic toxicity of the β-CD, would introduce a significant amount of free CD or free pesticide, influencing the experimental design.

The results are presented as LC50 values, which represent the concentration of each sample that causes 50% mortality in the exposed population, calculated along with their corresponding 95% confidence intervals (Table 1).

The results show that β-cyclodextrin, tested up to a concentration of 440.54 µM, did not induce 50% lethality in exposed rotifers, with a maximum observed effect of 21%. In contrast, the herbicides with the highest toxic activity towards *B. calyciflorus* were MON and PRO, showing LC50 values in the tens of micromoles/L. CLP was less toxic, with LC50 values in the range of hundreds of micromoles/L. Following complexation with β-cyclodextrin, all three herbicides exhibited a significant increase in toxicity, with LC50 values of the complexes reduced to a few units of micromoles/L. Notably, a marked enhancement of toxicity was observed upon complexation with β-cyclodextrin for all tested compounds. In particular, CLP exhibited the most pronounced effect, with a 36.4-fold increase in toxicity, as the LC50 decreased from 178.09 µM for the free herbicide to 4.89 µM for the β-CD complex, corresponding to only 2.75% of the original concentration (97.25% reduction). Similarly, MON showed a 12.7-fold increase in toxicity (LC50 reduced from 32.32 to 2.55 µM), representing 7.89% of the free compound LC50 (92.11% reduction). PRO also displayed a substantial enhancement, with a 9.07-fold increase in toxicity, as the LC50 decreased from 20.77 to 2.29 µM, corresponding to 11.03% of the initial concentration (88.97% reduction).

These results suggest that the increased toxicity of the herbicides following complexation with β-cyclodextrins (β-CD) may be related to an enhanced bioavailability of the active compounds in the test organism. Indeed, β-CD is known to increase the apparent aqueous solubility and dispersion of hydrophobic molecules, potentially increasing their concentration at the biological interface and facilitating their uptake across physiological barriers [31]. Consequently, a greater fraction of herbicide may become available to interact with cellular targets, resulting in enhanced toxic effects. β-CD itself may also contribute to toxicity through interactions with biological membranes. In fact, cyclodextrins are known to perturb membrane lipids, particularly cholesterol and, to a lesser extent, phospholipids, thereby influencing membrane structure and permeability.

Such membrane perturbations could exacerbate the toxic action of the herbicides and may also contribute to the ciliary damage observed both in vitro and in vivo [32,33].

**Figure 5:**
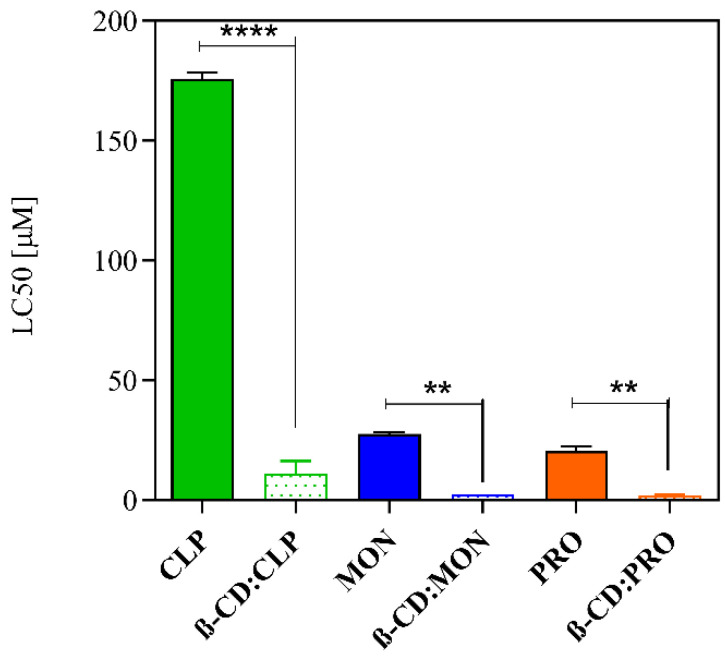
Acute toxicity of CLP, MON, and PRO and their corresponding β-CD inclusion complexes in *B. calyciflorus*. LC50s are expressed in µM as mean ± SD (n = 3). The significant difference compared to the control was determined using Unpaired *t*-test (**** *p* < 0.0001; ** *p* < 0.01).

To provide a more immediate visualization of the effects exerted by the herbicides on B. calyciflorus, the data were graphically depicted (Figure 5). Statistical analysis was performed using an Unpaired *t*-test to compare the LC50 values (expressed in micromolar units) of the herbicides in their free form with those of their respective β-cyclodextrin complexes.

What clearly emerges from Figure 5 is the marked difference between free CLP and its β-cyclodextrin complex (*p* < 0.0001). This effect is more pronounced than those observed for the β-cyclodextrin complexes of the other two herbicides (*p* < 0.01).

Direct comparison between the results of the present study and reported data in the literature is limited due to the scarce availability of ecotoxicological studies of the examined compounds on the selected aquatic organisms. Despite this gap, partial comparisons can still be drawn. For instance, Passananti et al. [34] reported an LC50 value of 164.70 µM for CLP in *B. calyciflorus*, in line with the results observed herein. In addition, tests conducted on *Thamnocephalus platyurus*, a planktonic crustacean belonging to the same trophic level as rotifers, revealed a greater sensitivity to CLP (LC50 = 47.55 µM). Regarding PRO, Villarroel et al. [35] observed that the cladoceran crustacean *Daphnia magna* exhibited significant mortality at concentrations equal to 2.5 µM. Conversely, to the best of current knowledge no recent data on the acute aquatic toxicity of MON appear to be available in the literature. However, since MON belongs to the phenylurea class of herbicides, reference to structurally similar compounds, such as diuron and linuron, can provide preliminary indications into its toxic effects. In particular, available data [36,37,38,39] indicate that the acute toxicity of diuron in secondary consumers as fish is comparable to that of linuron, with LC50s ranging from units to tens of micromoles per liter, aligning with the data presented in this research work.

No studies were found that specifically evaluate the effects of the selected herbicides when complexed with β-cyclodextrin. Nevertheless, regarding β-cyclodextrin, Lavorgna et al. [40] reported its low acute toxicity, with a maximum effect of 16.5% at 440.54 µM on *B. calyciflorus*, and an enhanced toxicity when β-cyclodextrin was complexed with imidacloprid, a neonicotinoid insecticide. The concordance between these data and the findings of the present study reinforce the hypothesis that, although β-cyclodextrin is poorly toxic in short-term exposure, it can significantly change the ecotoxicological behavior of pesticides once complexed, enhancing their toxicity.

#### 2.2.2. Chronic Toxicity Testing

In aquatic environments, organisms are often chronically exposed to sub-lethal pollutant concentrations, making long-term toxicity assessment essential. Chronic tests use concentrations at least ten times lower than acute assays, with exposure lasting about one-third of the organism’s life cycle better simulating real-world conditions.

For *R. subcapitata*, the endpoint was growth inhibition after 72 h of exposure; for *B. calyciflorus*, reproduction inhibition after 48 h of exposure. Results are expressed as EC50 values, representing the concentration causing a 50% effect (Table 2).

Table 2 shows that, in their free form, the three herbicides exhibited EC50 values of the same order of magnitude (µM) for both *R. subcapitata* and *B. calyciflorus*. However, for CLP, a significant difference was observed between the two species (*p* < 0.05), based on the EC50 confidence intervals. This difference may be attributed to the herbicide’s mode of action and the organisms’ biological characteristics. CLP inhibits mitosis by disrupting spindle formation [41], a process especially critical for microalgae. These findings align with those of Passananti et al. [34], who reported EC50 values of 2.01 µM for *R. subcapitata* and 10.95 µM for *B. calyciflorus*.

Regarding MON, Kathleen et al. [42] reported an EC50 of 533.60 µM in *D. magna* after 48 h of exposure. Although both *D. magna* and *B. calyciflorus* belong to the same trophic level (as primary consumers), the latter exhibited markedly higher sensitivity suggesting that trophic position alone does not adequately explain toxicological responses that are species-specific and thus depending on membrane permeability, metabolic rate, and detoxification capacity. For PRO, our results are consistent with those of Gómez de Barreda Ferraz et al. [43], who observed concentration-dependent growth inhibition in several freshwater phytoplankton species. Significant reductions in algal growth were reported at 0.69 µM for *Scenedesmus* spp. and 11.46 µM for *Chlorella* spp., with EC50 values ranging from single to tens of micromoles, partially overlapping with our findings for *R. subcapitata*. Chronic toxicity studies on *Daphnia* spp. by Villarroel and colleagues [35] and Pereira and colleagues [44] reported significant reductions in reproduction and feeding rates at sub-micromolar to low micromolar PRO concentrations. These effects are associated with interference in essential physiological pathways, including protein synthesis, energy metabolism, and environmental responsiveness [45]. Moreover, the bioaccumulative potential of PRO may exacerbate environmental stress, reducing long-term reproductive fitness in aquatic invertebrates (Pereira et al., 2007 [44]). Regarding the effects of β-cyclodextrin alone, they reveal differential sensitivity between organisms belonging to two trophic levels. In particular, primary consumers were more vulnerable than producers, as shown by the markedly lower EC50 value for *B. calyciflorus* (10.41 µM) compared to that of *R. subcapitata* (207.67 µM) (Table 2).

**Figure 6:**
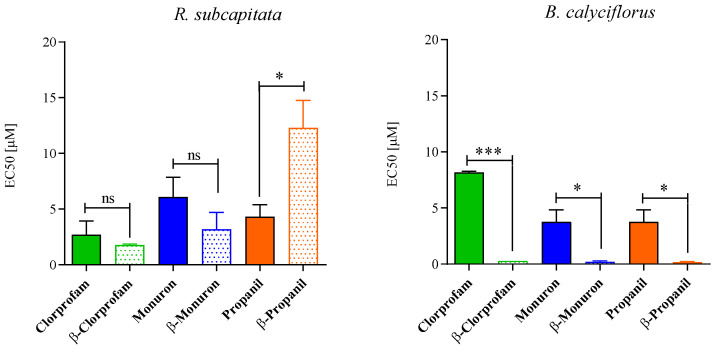
Chronic toxicity of CLP, MON, and PRO and their corresponding β-CD inclusion complexes *R. subcapitata* and *B. calyciflorus*. EC50 expressed in µM as mean ± SD (n = 3). Significant differences compared to the control were determined using the Unpaired test (* for *p* < 0.05 and *** for *p* < 0.0001, ns: no statistically significant difference).

This difference can be mainly attributed to structural distinctions between animal and plant cells. The rigid cellulose-based cell wall of *R. subcapitata* likely acts as a partial barrier to hydrophobic or high-molecular-weight molecules [46,47] as β-cyclodextrin, whereas *B. calyciflorus*, lacking such protection, is more directly exposed. In fact, in rotifers, a 48-h exposure to β-cyclodextrin induced alterations in corona cilia and compromised membrane integrity, impairing locomotion and feeding [32]. In addition, the findings reported herein are in line with Lavorgna et al. [16] who reported EC50 values of 223.79 µM for *R. subcapitata* and 13.57 µM for *B. calyciflorus.* When the selected herbicides were tested in complex with β-cyclodextrin (Table 2, Figure 6), distinct patterns emerged among the compounds. For β-CD:CLP and β-CD:MON, toxicity toward *R. subcapitata* remained relatively low and largely overlapped with that observed for the respective free herbicides, indicating that complexation did not substantially modify their effects. In contrast, a different behavior was observed for PRO: the β-CD:PRO led to a significant 65.52% reduction in EC50 values compared to free PRO.

In contrast, for the rotifer, all three herbicides showed a significant reduction in EC50 values when tested as complexes with β-CD (Table 3), with the most pronounced effect observed for CLP (*p* < 0.0001, Figure 6). Specifically, an increase in effect of 99.51% was recorded, suggesting that β-CD may act synergistically with the herbicide or enhance its bioavailability, thereby increasing long-term toxicity. The chronic toxicity study was further extended to the evaluation of EC20, EC10, and NOEC (Table 3) to better elucidate the toxicological differences between the free and complexed form of the herbicides. These parameters more closely reflect environmentally relevant concentrations [10,28,29].

The EC10 value is particularly important, as it represents the threshold below which no significant toxic effects are observed, while EC20 serves as an early indicator of toxicity, identifying the onset of measurable adverse effects. Considering the EC10 and EC20 values of β-cyclodextrin tested alone in *R. subcapitata* and *B. calyciflorus*, the rotifer once again exhibited greater sensitivity compared to the microalga.

For the alga, the EC20 and EC10 values were 37.18 µM and 13.04 µM, respectively, whereas for the rotifer they were 1.85 µM and 0.53 µM, respectively. These results are consistent with the findings of Lavorgna et al. [16]. Similar trends were observed for β-CD:herbicide; notably, the β-CD:MON showed a four-order of magnitude difference in EC20 between the alga (1.22 µM) and the rotifer (0.0002 µM). Among the free herbicides, MON and PRO were more toxic to the rotifer, whereas CLP exhibited greater toxicity toward the alga as previously reported by Passananti et al. [34].

The NOEC parameter can be used to assess environmental risk. Accordingly, the Risk Quotient (RQ) was calculated following standardized Environmental Risk Assessment (ERA) guidelines, such as the Technical Guidance Document (TGD, 2003). The RQ value is obtained from the ratio between MEC/PNEC. The MEC (Measured Environmental Concentration) represents the concentration detected in the environment and, in this case, corresponds to the most recent reported levels of the herbicides in freshwater environments worldwide: 0.68 ng/L for CLP, 325 ng/L for MON, 9000 ng/L for PRO [10,28,29].

The PNEC (Predicted No-Effect Concentration) was calculated by dividing the lowest NOEC value (Table 3) by a safety factor known as the Assessment Factor (AF), which depends on the number of trophic levels considered (TGD, 2003), and that for this research is 50. In addition, to facilitate the calculation, the NOEC values were converted to ng/L (2136 ng/L, 7946 ng/L, and 2181 ng/L for CLP, MON, and PRO, respectively). The resulting RQ for CLP was 0.01, representing no risk for the environment. In contrast, the RQ values calculated for MON and PRO RQ were higher than 1, suggesting a high risk for the aquatic environment. Furthermore, the environmental risk calculation was extended to the respective herbicide–β-cyclodextrin complexes.

Assuming a 1:1 stoichiometric ratio, the NOEC values determined for β-CD:CLP, β-CD:MON, and β-CD:PRO in rotifers were expressed as the equivalent concentration of the active herbicide present in solution and converted to ng/L for the calculation of RQ values. The RQ values calculated for β-CD:MON and β-CD:PRO were substantially higher than the safety threshold of 1 and exceeded those obtained for the corresponding free herbicides, indicating a high ecotoxicological risk associated with these complexes. In contrast, although the RQ calculated for β-CD:CLP was higher than that of the free herbicide, it remained consistently below the threshold value of 1 and therefore did not indicate environmental risk.

It should be noted that the ecotoxicological assessment and the resulting RQ estimates have been based on experiments conducted under controlled laboratory conditions, where the β-CD–herbicide complexes were prepared and tested in standardized media. Under environmentally realistic conditions, however, the stability and fate of these complexes may be influenced by several processes, including dissociation, dilution, competition with natural organic matter, and biotic and abiotic transformations. Therefore, the environmental exposure and toxicity of β-CD–herbicide complexes may differ from those observed under laboratory conditions, representing an important aspect that should be addressed in future studies.

## 3. Materials and Methods

### 3.1. Reagents

Chlorpropham (CLP, CAS: 101-21-3; C_10_H_12_ClNO_2_; MW: 213.66 g/mol), Monuron (MON, CAS:150-68-5; C_9_H_11_ClN_2_O; MW: 198.65 g/mol), Propanil (PRO, CAS: 709-98-8; CH_9_Cl_2_NO; MW: 218.08 g/mol) and β-cyclodextrin (β-CD, CAS: 7585-39-9; C_42_H_70_O_35_; MW: 1135 g/mol) were purchased from Sigma Aldrich (Milan, Italy).

### 3.2. Preparation of Herbicides- β-CD Inclusion Complexes

Inclusion complexes were prepared as previously described [26]. Herbicides and β-CD stock solutions in a 1:1 molar ratio were dissolved in phosphate buffer reaching the concentration of 0.05 mM and 5.0 mM respectively. The solutions of inclusion complexes were prepared mixing the stock solution with a 1:1 molar ratio. The pH values of the buffers at 7.1 were kept constant and continuously checked by using a calibrated pH-meter.

### 3.3. UV-Vis Spectroscopy Studies

All UV spectra were obtained using a UV-1700 Spectrometer (Shimadzu, Tokyo, Japan) with 1 cm matched quartz cuvettes. All measurements were recorded in the wavelength range 200–400 nm at room temperature.

#### 3.3.1. Determination of Molar Extinction Coefficients by UV-Vis Spectroscopy

Molar extinction coefficient (ε) was determined by adding, for each herbicide, an increasing concentration in buffered solution at pH 7.1. As the titration proceeded, resulting absorbance increase was monitored at specific wavelengths: 235 nm for CLP, 245 nm for MON, and 250 nm for PRO. Data were collected and fitted, using GraphPad 8 software, through the linear regression equation:

Y = *m*X + *b*

where Y is the absorbance value, *m* is the angular coefficient, X is herbicide concentration and *b* is the x-intercept. The slope of the line (*m*) is equal to the molar extinction coefficient (ε) of herbicide.

#### 3.3.2. Phase Solubility Studies by UV-Vis Spectroscopy

The solubility of herbicides is very low: 0.089 g/L for CLP, 0.240 g/L for MON and 0.13 g/L for PRO. An enhancement was valuated as the function of β-cyclodextrin concentrations. For each herbicide, a quantity five times higher than its solubility limit was added to 5 mL of aqueous solutions, increasing solubilizing agent concentration, in five round-bottom flasks. Specifically, stoichiometric molar ratios between guest and host in flasks (numbered from 1 to 5) were: 0:1; 0.25:1; 0.50:1; 0.75:1; and 1:1. Prior to each experiment, the pH of the herbicide stock solutions was measured, yielding values of 5.60 for CLP, 5.77 for MON, and 5.25 for PRO. Solutions in flasks were kept for 90 h under continuous stirring at 60 °C in thermic sand bath, using hot plates and stirring magnets. Before taking UV measurements, solutions were filtered with 0.22 nm filters. Filtered solutions were analyzed to determine the dissolved herbicide concentration.

Dissolved moles of herbicide in each solution were calculated with the following formula:

Mol = (Abs/ε) × volume

where “Abs” indicates absorbance value at specific wavelength for each herbicide, ε is the molar extinction coefficient and “volume” indicates the capacity of the cuvette used in the UV measurements.

Data were fitted, using GraphPad Software, through non-linear regression equation.

### 3.4. Solubility Studies by NMR Spectroscopy

All ^1^H-NMR experiments were carried out at 600 MHz using a Bruker AVANCE III HD 600 spectrometer (Billerica, MA, USA) equipped with a triple resonance Prodigy N2 cryoprobe having a z-axis pulse field gradient. The sample were prepared in H_2_O/D_2_O 90/10 (*v*/*v*) and the pH of aqueous solutions was the same as described for the phase solubility studies by UV-Vis. Various amounts of β-CD solution were added in fixed aliquots to a herbicide solution.

NMR experiments for collecting structural constraints were performed at 298 K referenced to external TMS (δ = 0 ppm).

Deuterium oxide (D_2_O) was purchased from Cambridge Isotope Laboratories (Andover, MA, USA). Mono (1D) spectra were accumulated with a spectral width of 5500 Hz.

For ^1^H-NMR experiments, a total of 32 scans were acquired using the Bruker 1D−^1^H pulse sequence (zgesgp), with a relaxation delay of 10 s between successive scans. Suppression of the residual water signal was achieved by excitation sculpting with pulsed field gradients. All spectra were recorded under identical experimental conditions to ensure comparability among samples. All the signals are integrated and compared between them.

### 3.5. Ecotoxicity

Stock solutions of herbicides and their complexes with β-cyclodextrin ([CLP]= 468 µM, [MON]= 503 µM, [PRO] = 458 µM, [β-CD] = 881 µM, [β-CD:CLP] = 74 µM, [β-CD:MON] = 75 µM, [β-CD:PRO] = 74 µM) were prepared using Milli-Q water. Fresh test solutions were prepared immediately before use by diluting the stocks into standardized synthetic media, specifically formulated to meet the physiological requirements of the target test organism.

#### 3.5.1. Acute Toxicity Testing

Cysts of the rotifer *B. calyciflorus* (MicroBioTest Inc., Nazareth, Belgium) were hatched in synthetic freshwater (pH 7.5 ± 0.3; hardness: 80–100 mg/L CaCO_3_) in Petri dishes maintained at 25 ± 1 °C under continuous illumination (3000–4000 lux) for 16–18 h (ASTM E1440-91) [48]. The herbicides CLP, MON, and PRO were tested, after conversion of molecular weight from mg/L to µM to allow an easier comparison among molecules, and concentrations ranged from 1.83 to 234.02 µM, 1.97 to 251.70 µM, and 1.79 to 229.27 µM, respectively. β-CD, β-CD:CLP, β-CD:MON, and β-CD:PRO were evaluated within the range of 13.76–440.54 µM, 0.29–37.07 µM, 0.29–37.49 µM, and 0.29–36.95 µM, respectively, using a serial dilution factor of 2. Bioassays were conducted in 36-well plates, each well containing 0.3 mL of the test solution or synthetic freshwater (negative control), and five rotifers, with six replicates per concentration. Plates were incubated in the dark at 25 °C for 24 h. Results were expressed as the median lethal concentration (LC50), defined as the concentration causing 50% mortality of the exposed organisms.

#### 3.5.2. Chronic Toxicity Testing

Chronic toxicity assessments were conducted using the green alga *R. subcapitata* as a model primary producer and the rotifer *B. calyciflorus* as a representative consumer. The algal test was conducted in accordance with OECD Guideline 201 (2011), incorporating minor adaptations as described by Nugnes and coauthors [30]. The rotifer assay followed the procedures outlined in ISO 20666 (2008) [49].

In the algal growth inhibition test, each well of a 96-well plate received 0.2 mL of the test solution and 0.1 mL of an algal inoculum containing 10^4^ cells/mL. Concentrations were selected based on preliminary range-finding studies and prepared using a dilution factor of 3.2, within ranges of 0.01–46.80 µM, 0.01–161.09 µM, 0.44–146.73 µM for CLP, MON and PRO, respectively, and within the ranges of 0.41–440.54 µM, 0.23–23.73 µM, 0.23–23.99 µM, and 2.31–23.65 µM for β-CD, β-CD:CLP, β-CD:MON, and β-CD:PRO, respectively, using a dilution factor of 3.2. Plates were incubated at 25 ± 1 °C under continuous illumination ranging at 6000 lux. Algal growth was monitored by measuring absorbance at 450 nm at 0, 24, 48, and 72 h using a microplate reader (Synergy H1, Biotek, Winooski, VT, USA).

Rotifer hatching followed the ISO procedure. Organisms less than 2 h post-hatch were transferred to 48-well plates, each containing 0.9 mL of the test solution and 0.1 mL of *R. subcapitata* culture (10^7^ cells/mL), along with one neonate. Incubation was performed in the dark at 25 ± 1 °C for 48 h. Exposure concentrations, determined via range-finding test, were within ranges of 0.01–46.80 µM, 0.01–50.34 µM, and 0.01–45.85 µM for CLP, MON and PRO, respectively, and within the ranges of 0.03–88.11 µM, 0.001–7.41 µM, 0.002–7.50 µM, and 0.002–7.40 µM for β-CD, β-CD:CLP, β-CD:MON, and β-CD:PRO, respectively, using a dilution factor of 3.2. Negative controls consisted of organisms maintained in synthetic medium only.

The chronic endpoints evaluated reproductive inhibition in rotifers and algal growth inhibition, with toxicological thresholds reported as effective concentration values (ECX).

### 3.6. Toxicity Data Analysis

Three independent experiments were conducted for each toxicity test, and the lethal/effect percentages were combined to determine the concentrations causing x% lethality/effect using non-linear regression (log agonist vs. normalized response-variable slope—Prism 8 software, GraphPad Inc., San Diego, CA, USA). The No Observed Effect Concentration (NOEC) was assessed through ANOVA and Dunnett’s multiple comparison test by identifying significant differences from the negative control (* *p* < 0.05, ** *p* < 0.001, *** *p* < 0.001). The Unpaired *t*-test was used to compare the effects induced by individual herbicides with those produced by their respective β-cyclodextrin inclusion complexes (* *p* < 0.05, ** *p* < 0.001, and *** *p* < 0.0001).

## 4. Conclusions

This study provides a comprehensive evaluation of the solubility enhancement and aquatic ecotoxicity of three herbicides, chlorpropham, monuron and propanil, when included in β-cyclodextrin complexes.

The solubility enhancement due to the complexation of the herbicides studied, reported by the phase solubility diagrams, is in agreement with the NMR data. In particular, the A_L_-type phase solubility diagrams confirm the1:1 stoichiometry reported in Dragone et al. 2023 [26].

However, this confirmed improvement in post-complexation solubility for the three herbicides cannot be considered entirely beneficial. Indeed, ecotoxicological tests demonstrate that complexation with β-CD substantially modifies their toxicological profiles. Acute toxicity assays performed with *B. calyciflorus* showed a marked increase in toxicity after complexation, with LC50 values decreasing by approximately one order of magnitude for all compounds. This effect is likely associated with the increased solubility and bioavailability of the active substances, which may facilitate their transport across biological membranes and increase organism exposure. Chronic toxicity assays further highlighted species-specific responses. In particular, the rotifer *B. calyciflorus* exhibited consistently higher sensitivity to the β-CD:herbicide complexes, with a pronounced reduction in EC50 values, indicating that cyclodextrin complexation may enhance long-term toxicity in aquatic primary consumers.

Environmental Risk Assessment (ERA), conducted according to ERA guidelines and the Technical Guidance Document (TGD, 2003), indicated negligible risk for CLP under currently reported environmental concentrations, whereas MON and PRO exceeded the safety threshold (RQ > 1). Notably, the corresponding β-CD inclusion complexes showed even higher RQ values, suggesting that cyclodextrin-based formulations may increase the environmental risk of these herbicides despite improving their solubility and delivery.

These findings highlight that, although cyclodextrin complexation represents a promising strategy for enhancing the physicochemical properties of poorly soluble pesticides, its ecological implications must be carefully considered. Therefore, ecotoxicological evaluation of cyclodextrin-based formulations should be systematically incorporated into environmental risk assessment frameworks, particularly with regard to non-target aquatic organisms that are vulnerable to agricultural runoff.

Future research should focus on the development of safer herbicide delivery systems following a safe-by-design approach. Promising alternatives include biodegradable and controlled-release carriers such as chitosan-, alginate-, cellulose-, starch-, guar gum-, and lignin-based matrices, hydrogels, and clay–biopolymer composites. These systems may improve formulation performance while reducing environmental exposure peaks and limiting excessive increases in bioavailability. However, their ecotoxicological profiles should be assessed both in combination with the active ingredient and separately through carrier-only controls to ensure that enhanced delivery efficiency is not accompanied by unintended adverse effects on aquatic ecosystems.

## Figures and Tables

**Table 1:** Acute toxicity of β-CD, CLP, MON and PRO and their corresponding β-CD inclusion complexes in *B. calyciflorus*. LC50 values are expressed in µM with a 95% confidence interval.

	β-CD	CLP	β-CD:CLP	MON	β-CD:MON	PRO	β-CD:PRO
*B. calyciflorus*	n.d. *	178.09 (139.57–227.23)	4.89 (2.83–8.43)	32.32 (26.58–39.31)	2.55 (1.75–3.71)	20.77 (17.24–24.99)	2.29 (1.64–3.21)

n.d. *: percentage of effect equal to 21.07 ± 5.08 at 440.54 µM.

**Table 2:** EC50 values (95% confidence intervals) expressed in µM of chronic toxicity of β-CD, CLP, MON, and PRO and their corresponding β-CD inclusion complexes in *R. subcapitata* and *B. calyciflorus*.

	β-CD	CLP	β-CD:CLP	MON	β-CD:MON	PRO	β-CD:PRO
*R. subcapitata*	207.67 (169.34–254.81)	2.57 (1.92–3.56)	1.76 (1.42–2.19)	6.39 (4.33–9.36)	2.91 (1.42–5.95)	4.22 (3.03–5.82)	12.24 (8.73–17.15)
*B. calyciflorus*	10.41 (7.72–14.12)	8.19 (6.50–10.30)	0.04 (0.03–0.07)	3.27 (2.16–5.01)	0.02 (0.01–0.03)	3.44 (2.15–5.41)	0.09 (0.06–0.16)

**Table 3:** EC20, EC10, NOEC, and LOEC values expressed in µM, obtained from the chronic toxicity of β-CD, CLP, MON, and PRO and their corresponding β-CD inclusion complexes in *R. subcapitata* and *B. calyciflorus*.

	β-CD		CLP		β-CD:CLP		MON		β-CD:MON		PRO		β-CD:PRO	
	*R. subcapitata*	*B. calyciflorus*	*R. subcapitata*	*B. calyciflorus*	*R. subcapitata*	*B. calyciflorus*	*R. subcapitata*	*B. calyciflorus*	*R. subcapitata*	*B. calyciflorus*	*R. subcapitata*	*B. calyciflorus*	*R. subcapitata*	*B. calyciflorus*
**EC20**	33.15 (24.26–43.54)	1.36 (0.82–2.14)	0.56 (0.33–0.89)	1.26 (0.84–1.78)	0.61 (0.50–0.75)	4 × 10^−4^ (2 × 10^−4^–9 × 10^−4^)	2.01 (1.06–3.42)	0.30 (0.15–0.55)	1.31 (0.50–2.98)	2 × 10^−4^ (1 × 10^−4^–6 × 10^−4^)	1.88 (1.10–2.93)	0.37 (0.18–0.69)	6.68 (4.30–10.01)	7 × 10^−4^ (3 × 10^−4^–2 × 10^−3^)
**EC10**	11.33 (7.18–17.54)	0.41 (0.21–0.81)	0.23 (0.14–0.42)	0.42 (0.23–0.70)	0.33 (0.25–0.44)	3 × 10^−5^ (2 × 10^−5^–8 × 10^−5^)	1.06 (0.50–2.26)	0.07 (0.02–0.18)	0.82 (0.29–2.36)	2 × 10^−5^ (4 × 10^−6^–9 × 10^−5^)	1.15 (0.60–2.25)	0.09 (0.04–0.27)	4.69 (2.84–8.12)	4 × 10^−5^ (2 × 10^−5^–1 × 10^−4^)
**NOEC**	6.87	0.08	0.01	0.14	0.22	0.0002	1.51	0.04	0.67	0.0001	0.41	0.01	2.29	0.0002

## Data Availability

Data will be made available on request.
